# Gallbladder volvulus

**DOI:** 10.1002/ccr3.5549

**Published:** 2022-04-08

**Authors:** Zeinab AbdelMohsen Abdelrahman, Razana Mohd Omar, Salman Mirza, Süha Türkmen

**Affiliations:** ^1^ 36977 Department of Emergency Medicine Hamad Medical Corporation Doha Qatar; ^2^ 36977 Department of Radiology Hamad Medical Corporation Doha Qatar; ^3^ Department of Emergency Medicine Acibadem University Istanbul Turkey

**Keywords:** emergency, Gallbladder, ultrasound, volvulus

## Abstract

Gallbladder volvulus is a rare entity. The condition results in rotation of the gallbladder on its mesentery along the axis of cystic duct and artery. Gallbladder volvulus is a condition in which the organ twists on its long axis to the point where its vascular supply is compromised.

## Graphical abstract

Gallbladder volvulus is a rare entity which mostly occurs in elderly, but it might occur in young patients as well. Ultrasound is safe and as sensitive as MRI in diagnosis of gallbladder volvulus.

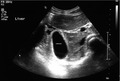

## 1 CLINICAL IMAGE

**FIGURE 1:**
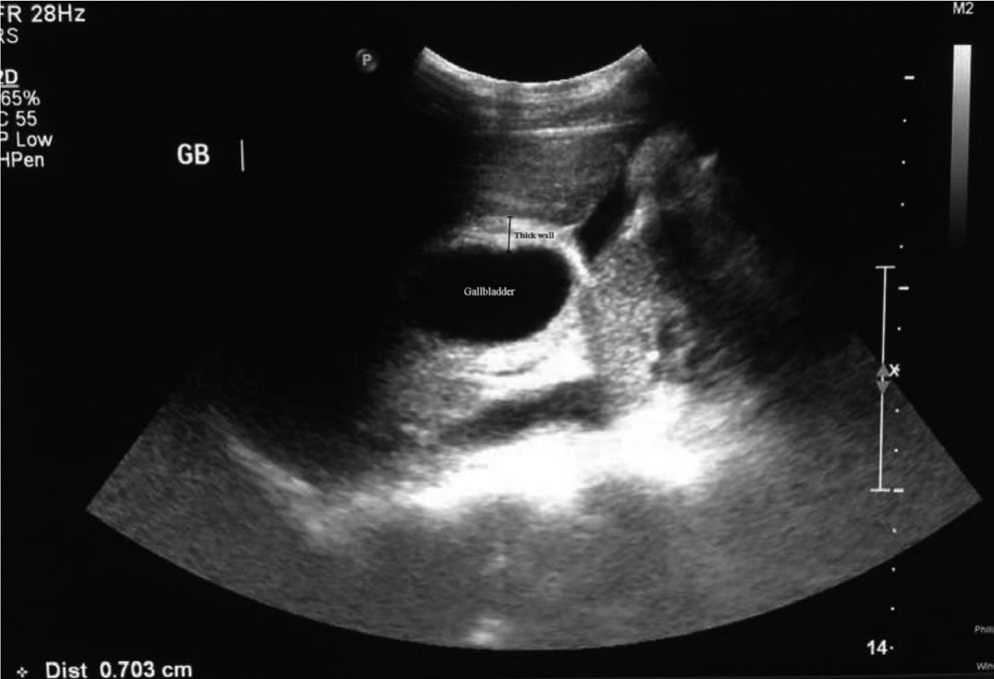
Ultrasound appearance of gallbladder volvulus: note significant gallbladder wall thickness without stones

**FIGURE 2:**
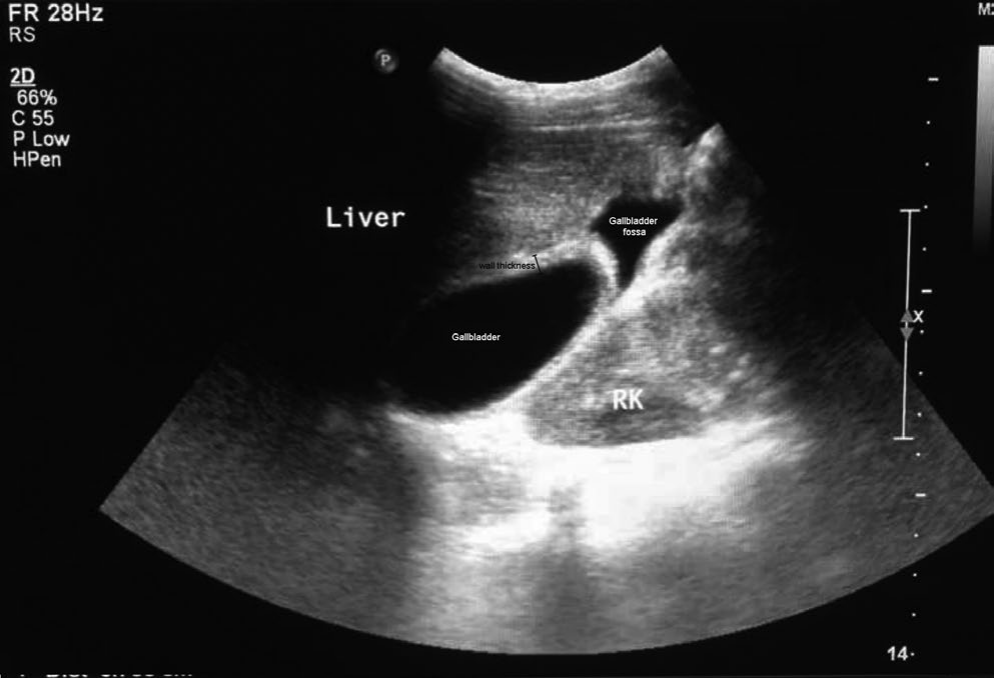
Ultrasound appearance of gallbladder volvulus: gallbladder fossa marked with an arrow (↑), note that gallbladder is floating out of fossa/anterior

**FIGURE 3:**
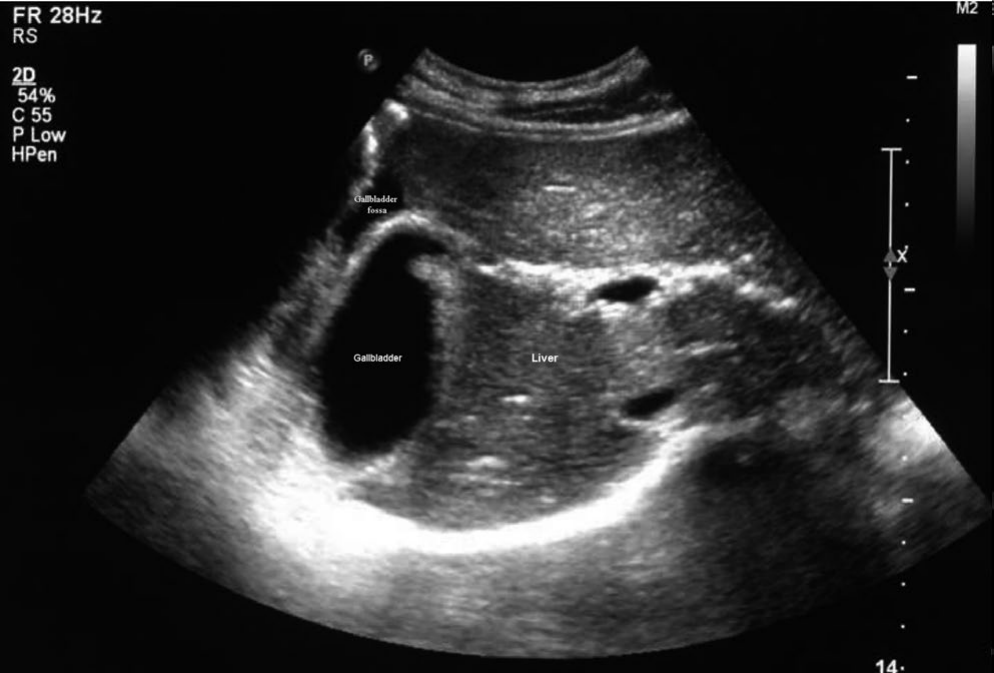
Ultrasound appearance of gallbladder volvulus

**FIGURE 4:**
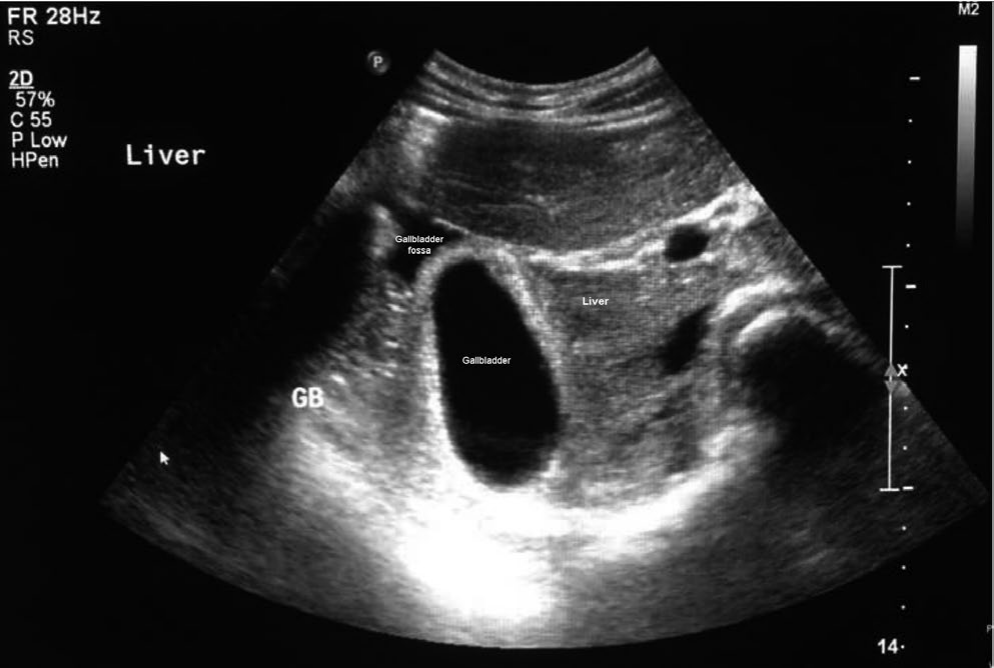
Gallbladder fossa marked with an arrow (↑), note that gallbladder is not in its normal anatomical position

An 18‐year‐old female patient presents to emergency department with right upper quadrant pain for 1 day, associated with repeated vomiting. The patient had a tender at the right hypochondrium. The patient was evaluated as a case of acute cholecystitis and started on intravenous fluids and analgesics. Laboratory investigations revealed as normal, ultrasound showed that the gallbladder was out of the fossa with significant edema and wall thickness (Figures 1, 2, 3, and 4).

## 2 DIAGNOSIS

Gallbladder volvulus is a condition in which the organ twists on its long axis to the point where its vascular supply is compromised. Anatomical abnormalities can result in a gallbladder that is suspended on an abnormally long mesentery that allows it to hang freely from the liver bed and consequently making it more susceptible to rotational instability. Complete torsion causes an acute presentation, and an incomplete one is usually associated with recurrent episodes of slowly progressing pain.^1^ The usual diagnostic modalities are ultrasound and contrast‐enhanced CT. MRCP can also aid the diagnosis, showing a V‐shaped distortion of extrahepatic bile ducts due to traction by the cystic duct. Prompt detorsion and cholecystectomy are mandatory to avert the potentially fatal sequelae of gangrene and perforation.^2^

## CONFLICT OF INTEREST

None declared.

## AUTHOR CONTRIBUTION

Zeinab Abdel Mohsen Abdelrahman and Razana Mohd Omar conceived and drafted the manuscript. Salman Mirza and Suha Turkmen reviewed the literature.

## CONSENT

Written informed consent was obtained from the patient to publish this report in accordance with the journal's patient consent policy.

## Data Availability

The data that support the findings of this study are available on request from the corresponding author. The data are not publicly available due to privacy or ethical restrictions.
